# Exogenously Scavenged and Endogenously Synthesized Heme Are Differentially Utilized by Mycobacterium tuberculosis

**DOI:** 10.1128/spectrum.03604-22

**Published:** 2022-09-28

**Authors:** Rebecca K. Donegan, Yibo Fu, Jacqueline Copeland, Stanzin Idga, Gabriel Brown, Owen F. Hale, Avishek Mitra, Hui Yang, Harry A. Dailey, Michael Niederweis, Paras Jain, Amit R. Reddi

**Affiliations:** a School of Chemistry and Biochemistry, Georgia Institute of Technology, Atlanta, Georgia, USA; b School of Biological Sciences, Georgia Institute of Technology, Atlanta, Georgia, USA; c Parker Petit Institute for Bioengineering and Biosciences, Georgia Institute of Technology, Atlanta, Georgia, USA; d Department of Microbiology, University of Georgia, Athens, Georgia, USA; e Department of Biochemistry and Molecular Biology, University of Georgia, Athens, Georgia, USA; f Department of Microbiology, University of Alabama at Birmingham, Birmingham, Alabama, USA; g Department of Microbiology and Immunology, Albert Einstein College of Medicine, Bronx, New York, USA; h Department of Neurology, Albert Einstein College of Medicine, Bronx, New York, USA; i Department of Pathology, Laura and Isaac Perlmutter Cancer Center, New York University Grossman School of Medicine, New York, New York, USA; j Department of Chemistry, Barnard College, New York, New York, USA; k Cell Therapy and Cell Engineering Laboratory, Memorial Sloan Kettering Cancer Center, New York, New York, USA; Johns Hopkins University School of Medicine

**Keywords:** *Mycobacterium tuberculosis*, *Mycobacterium smegmatis*, heme homeostasis, iron homeostasis, heme synthesis, heme, heme sensors, heme transport, iron, tuberculosis

## Abstract

Heme is both an essential cofactor and an abundant source of nutritional iron for the human pathogen Mycobacterium tuberculosis. While heme is required for M. tuberculosis survival and virulence, it is also potentially cytotoxic. Since M. tuberculosis can both synthesize and take up heme, the *de novo* synthesis of heme and its acquisition from the host may need to be coordinated in order to mitigate heme toxicity. However, the mechanisms employed by M. tuberculosis to regulate heme uptake, synthesis, and bioavailability are poorly understood. By integrating ratiometric heme sensors with mycobacterial genetics, cell biology, and biochemistry, we determined that *de novo*-synthesized heme is more bioavailable than exogenously scavenged heme, and heme availability signals the downregulation of heme biosynthetic enzyme gene expression. Ablation of heme synthesis does not result in the upregulation of known heme import proteins. Moreover, we found that *de novo* heme synthesis is critical for survival from macrophage assault. Altogether, our data suggest that mycobacteria utilize heme from endogenous and exogenous sources differently and that targeting heme synthesis may be an effective therapeutic strategy to treat mycobacterial infections.

**IMPORTANCE** Mycobacterium tuberculosis infects ~25% of the world’s population and causes tuberculosis (TB), the second leading cause of death from infectious disease. Heme is an essential metabolite for M. tuberculosis, and targeting the unique heme biosynthetic pathway of M. tuberculosis could serve as an effective therapeutic strategy. However, since M. tuberculosis can both synthesize and scavenge heme, it was unclear if inhibiting heme synthesis alone could serve as a viable approach to suppress M. tuberculosis growth and virulence. The importance of this work lies in the development and application of genetically encoded fluorescent heme sensors to probe bioavailable heme in M. tuberculosis and the discovery that endogenously synthesized heme is more bioavailable than exogenously scavenged heme. Moreover, it was found that heme synthesis protected M. tuberculosis from macrophage killing, and bioavailable heme in M. tuberculosis is diminished during macrophage infection. Altogether, these findings suggest that targeting M. tuberculosis heme synthesis is an effective approach to combat M. tuberculosis infections.

## INTRODUCTION

Heme is both an essential cofactor and a potential source of iron for the human pathogen Mycobacterium tuberculosis (1, 2). As a cofactor, heme enables many physiological functions, including respiration, gas sensing, and protection against reactive oxygen and nitrogen species generated by the host immune system. Nutritionally, heme is the most bioavailable source of iron in the human host, with more than two-thirds of iron in circulation bound to hemoglobin as heme-iron (3). Although heme is essential for M. tuberculosis, which can both make and scavenge heme, it is also potentially cytotoxic if present in excess or mishandled by cells (4, 5). Consequently, M. tuberculosis must tightly regulate heme synthesis, import, and bioavailability to mitigate heme toxicity. However, the mechanisms underlying the regulation of heme homeostasis in mycobacteria are poorly understood (1, 6, 7).

**FIG 1:**
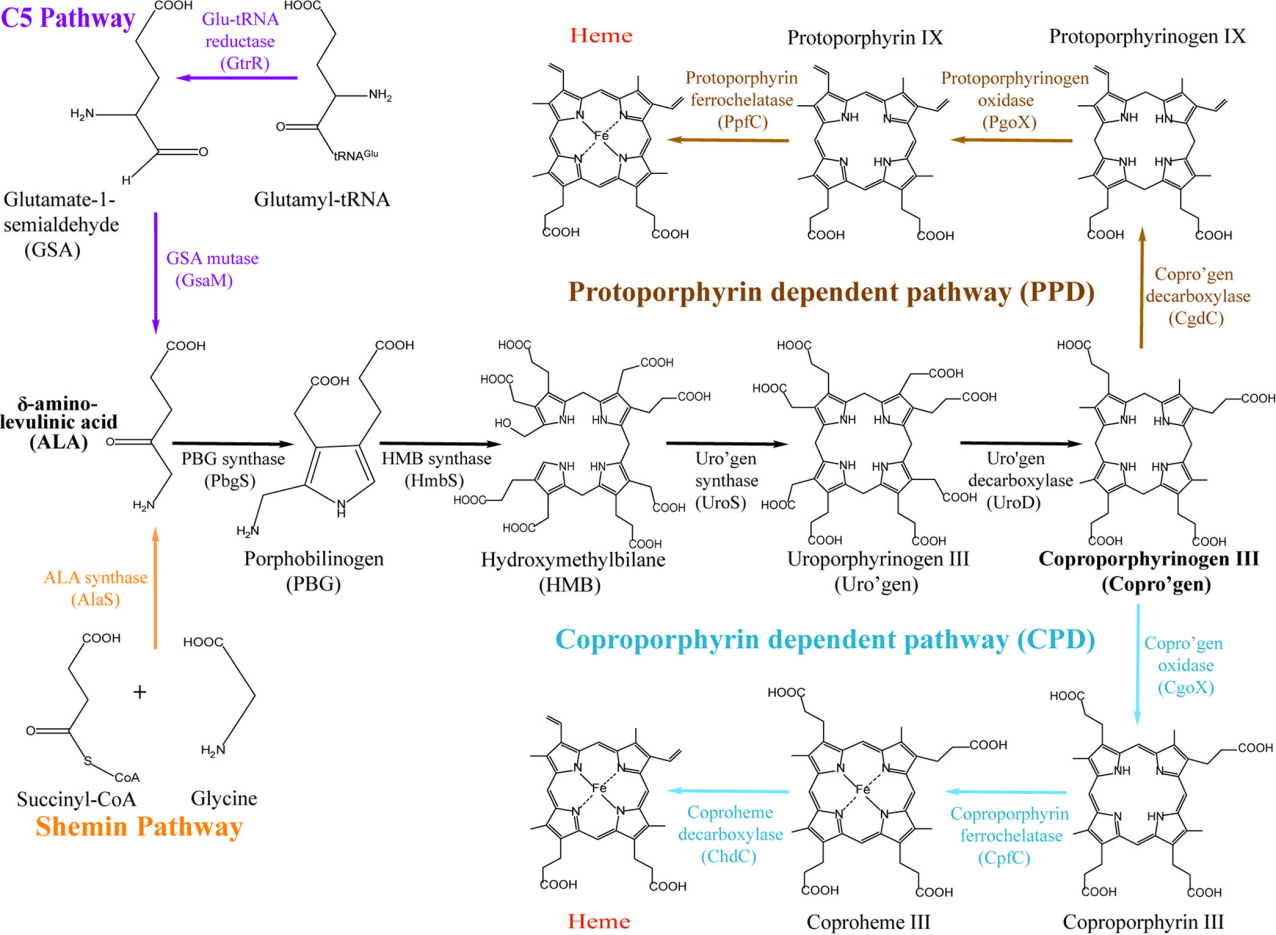
The protoporphyrin-dependent (PPD) and coproporphyrin-dependent (CPD) heme biosynthesis pathways. Enzymes involved in aerobic heme biosynthesis via the PPD and CPD pathways are shown. Heme biosynthesis begins with the formation of aminolevulinic acid (ALA). Animals, fungi, and some bacteria use the Shemin pathway (C4 pathway), where succinyl CoA and glycine are condensed to make ALA. Plants and most bacteria utilize the C5 pathway, which makes ALA reductively from glutamyl-tRNA. ALA is subsequently condensed, dehydrated, and decarboxylated to form coproporphyrinogen III. The PPD pathway is used by Gram-negative bacteria and eukaryotes to make heme from coproporphyrinogen. The CPD pathway is found mostly in the Gram-positive *Firmicutes* and *Actinobacteria*. Mycobacteria utilize the C5 pathway and the CPD pathway for heme biosynthesis.

M. tuberculosis encodes a complete heme biosynthetic pathway via the coproporphyrin-dependent (CPD) branch (8–10). The CPD branch diverges from the canonical protoporphyrin-dependent (PPD) branch used by eukaryotes and Gram-negative bacteria at the three terminal heme synthesis steps (Fig. 1). All heme-synthesizing organisms produce aminolevulinic acid (5-ALA), which is ultimately condensed, dehydrated, and decarboxylated to form coproporphyrinogen III (Fig. 1). In the PPD branch, coproporphyrinogen III is first oxidatively decarboxylated to protoporphyrinogen III, then oxidized to protoporphyrin IX, and then, finally, metallated with iron to make heme (Fig. 1). In contrast, in the CPD pathway, coproporphyrinogen III is first oxidized to coproporphyrin III, then metallated with iron to make coproheme, and then oxidatively decarboxylated to make heme. The divergence of heme synthesis in M. tuberculosis and the necessity of heme for M. tuberculosis survival has led to the consideration of targeting heme synthesis for anti-M. tuberculosis therapies (6, 9, 11). However, since M. tuberculosis can also scavenge exogenous heme, the impact of inhibiting *de novo* heme synthesis during infection is unclear.

Exogenous heme can be utilized both as an iron source (7, 12, 13) and as a cofactor for hemoproteins as evidenced by the rescue of a Δ*cpfC* deletion mutant lacking ferrochelatase by heme (Fig. 1 and Table S1 in the supplemental material) (14). Heme can be scavenged by secreted hemophores such as Rv0203 (15, 16) or via cell surface heme binding and transport proline-proline-glutamate (PPE) proteins (7, 17). Moreover, in the presence of albumin, heme uptake can occur without the need for the PPE heme binding proteins, suggesting the presence of both albumin-dependent and -independent heme uptake pathways (7, 12, 13). The presence of multiple pathways for heme scavenging, along with reduced survival of M. tuberculosis in macrophages when heme uptake is disrupted, (12) implies that heme scavenging is important for M. tuberculosis virulence. It is unknown how heme uptake is regulated and how its metabolism is coordinated to satisfy the need of M. tuberculosis for heme as a cofactor and for iron.

Here, by integrating genetically encoded ratiometric heme sensors with mycobacterial molecular genetics and biochemical assays, we probe the coordination of heme uptake, synthesis, and bioavailability in M. tuberculosis and Mycobacterium smegmatis. Our results establish that both M. tuberculosis and M. smegmatis maintain a reservoir of exchangeable bioavailable heme and that *de novo*-synthesized heme is more bioavailable than exogenously supplied heme. Moreover, while exogenous heme downregulates the expression of heme biosynthesis genes early in the pathway (*gtrR* and *gsaM*, Fig. 1), heme deficiency, it does not induce expression of known heme uptake genes. We further show that *de novo* heme synthesis contributes to survival of M. tuberculosis in macrophages. Altogether, our data suggest that targeting heme synthesis in mycobacteria may be an effective strategy to treat mycobacterial infections.

## RESULTS

### Characterization of heme bioavailability in M. smegmatis and M. tuberculosis.

**FIG 2:**
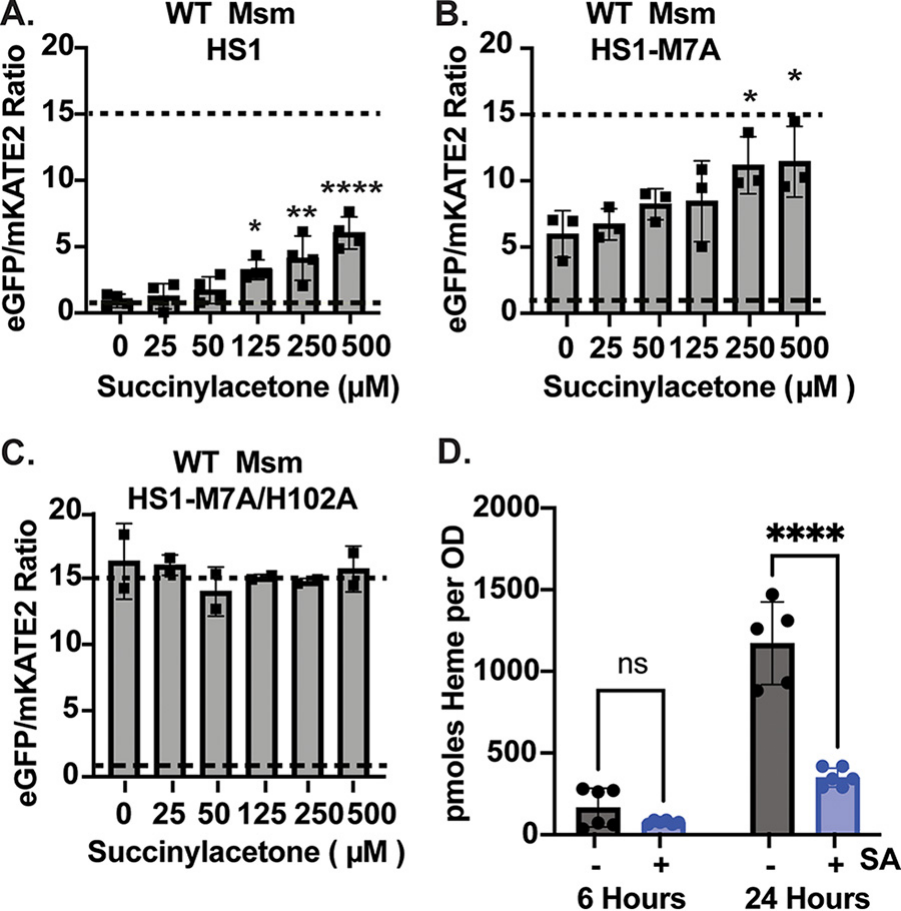
Characterization of heme sensors in M. smegmatis. (A to C) The eGFP/mKATE2 fluorescence ratio of the heme sensors HS1 (A), HS1-M7A (B), and HS1-M7A/H102A (C) in M. smegmatis as a function of the concentration of the heme synthesis inhibitor succinylacetone (SA). (D) Total cellular heme in WT M. smegmatis and WT M. smegmatis treated with 500 μM SA. Data in panels A to C represent the mean ± standard deviation (SD; error bars) for *n* = 3. In panel D, *n* = 5 (24 h, no SA) or 6 (all others). In panels A to C, the statistical significance was assessed by one-way analysis of variance (ANOVA) with Dunnett’s *post hoc* test using untreated M. smegmatis (0 μM SA) as the reference: (A) *, *P = *0.0264; **, *P = *0.0023; ****, *P* < 0.0001. (B) *, *P = *0.0417 and 0.0316. In panel D, the statistical significance was assessed by two-way ANOVA with a *post hoc* Šídák’s multiple-comparison test. ns, *P = *0.4740; ****, *P* < 0.0001.

**FIG 3:**
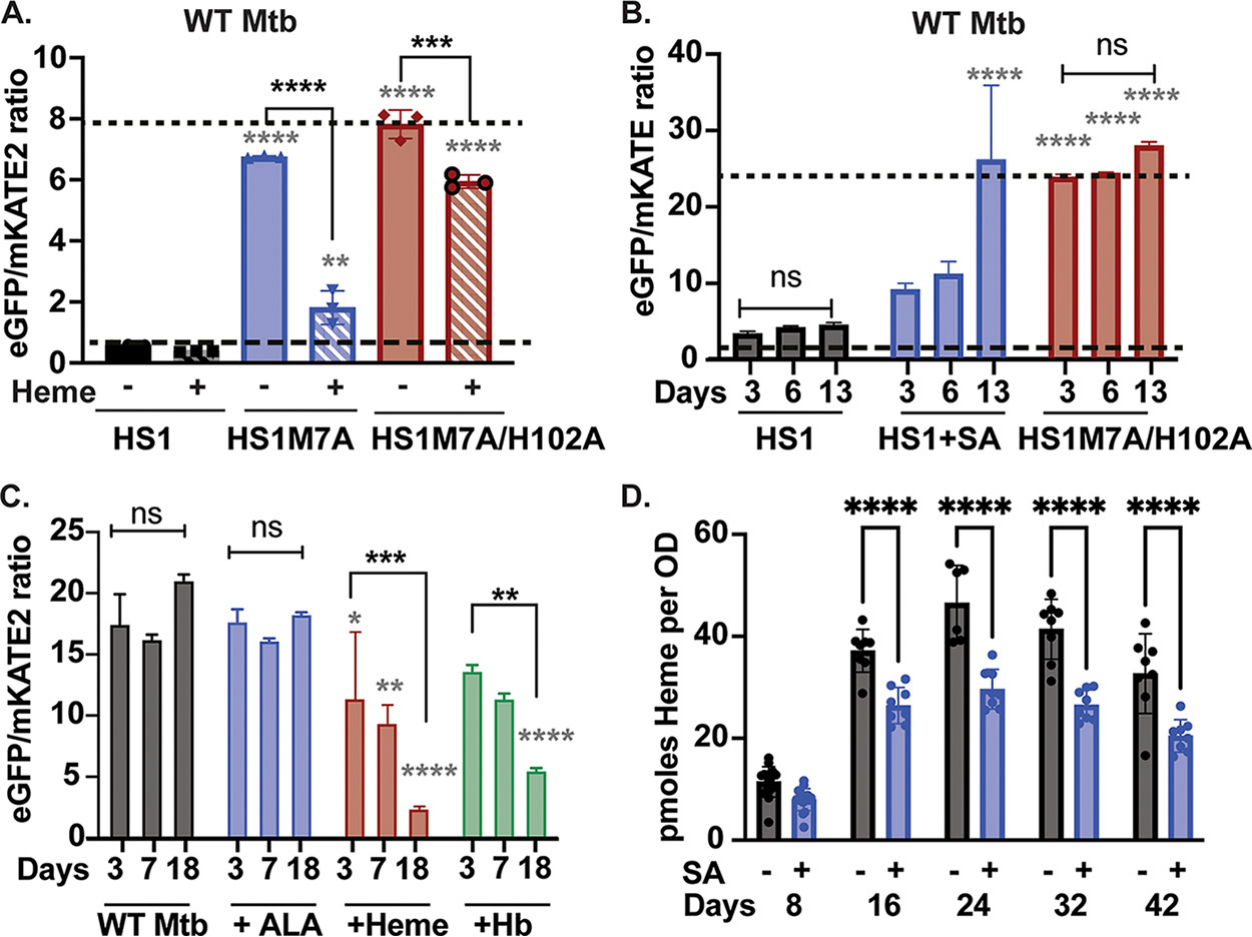
Characterization of heme sensors in M. tuberculosis. (A) Fluorescence ratios of the indicated heme sensors expressed in WT M. tuberculosis cells conditioned with or without 25 μM hemin chloride for 13 days. (B) Fluorescence ratio of HS1 expressed in WT M. tuberculosis cells conditioned with or without 500 μM SA over time. (C) Time-dependent fluorescence ratio of HS1-M7A expressed in WT M. tuberculosis cells conditioned with 5 μg/mL of the heme precursor 5-aminolevulinic acid (ALA), 25 μM hemin chloride, or 6.25 μM hemoglobin (Hb). In panels A and B, fluorescence ratios indicative of 0% and 100% heme bound to sensor as determined by sensor calibration experiments are denoted by small and large dashed lines, respectively. (D) Concentration of total cellular heme in WT M. tuberculosis and WT M. tuberculosis treated with 500 μM SA. All error bars shown are the SD of *n* = 3 in panels A to C, *n* = 8 in panel D, and *n* = 16 at 8 h in panel D. In panel A, the statistical significance was assessed by one-way ANOVA with Šídák’s multiple-comparison test to compare heme-treated and untreated cells for each sensor. Black asterisks indicate significant differences between heme-treated and untreated M. tuberculosis cells: (HS1) *P = *0.8353; (HS1-M7A) ****, *P < *0.0001; (HS1-M7A/H102A) ***, *P = *0.0001. Gray asterisks denote significant differences between HS1 and the indicated heme sensor in cells cultured with matched amounts of heme: ****, *P < *0.0001; **, *P = *0.0015. In panel B, the statistical significance was assessed by two-way ANOVA with a Bonferroni *post hoc* test. Black asterisks denote significant differences relative to day 3 for each sensor or growth condition: ***, *P = *0.0002; ns, nonsignificant differences, with *P = *0.6681 and > 0.9999. Gray asterisks denote significant differences relative to HS1 for a given day: ****, *P < *0.0001. In panel C, the statistical significance was assessed by two-way ANOVA with a Bonferroni *post hoc* test. Black asterisks denote significant differences relative to day 3 for each growth condition: ***, *P = *0.0004; **, *P = *0.0029; ns, nonsignificant differences and *P > *0.9999 and *P = *0.0881 for day 3 versus day 18 in untreated and ALA-treated samples, respectively. Gray asterisks denote significant differences relative to the WT for each time point: *, *P = *0.0358; **, *P = *0.0087, ****, *P < *0.0001. In panel D, the statistical significance was assessed by using a two-way ANOVA with a *post hoc* Šídák’s multiple-comparison test between treated and untreated samples at each time point. Day 8 *P* value = 0.1281; for other time points ****, *P < *0.0001. Unless otherwise noted, differences that are not statistically significant are unlabeled.

Total cellular heme is the sum of exchange inert and labile heme (1, 4, 5, 18). The majority of intracellular heme is tightly bound to hemoproteins and exchange inert. A smaller fraction of intracellular heme is kinetically labile, readily exchanging between various biomolecules, and is bioavailable for heme-dependent processes. To probe labile heme within mycobacteria, we incorporated and validated previously described genetically encoded labile heme sensors (18–22) in M. smegmatis and M. tuberculosis (Fig. 2 and 3). Heme sensor 1 (HS1) is a tri-domain construct consisting of the heme binding protein cytochrome *b*_562_ (Cyt *b*_562_), fused to enhanced green fluorescent protein (eGFP), and monomeric Katushka red fluorescent protein 2 (mKATE2). The fluorescence of eGFP is quenched upon heme binding to Cyt *b*_562_, whereas the mKATE2 fluorescence is unaffected (18). Thus, the eGFP/mKATE2 fluorescence ratio is inversely proportional to cellular labile heme (18).

Since labile heme may vary between cells and organisms, we developed sensors with different heme affinities to ensure precise and accurate measurements of labile heme. The prototype heme sensor binds heme with high affinity using methionine 7 and histidine 102 within the Cyt *b*_562_ domain of HS1, which exhibits dissociation constants of 3 nM for ferric heme [*K*_D_^Fe(III)^] and 1 pM for ferrous heme [*K*_D_^Fe(II)^] (18, 19). Mutation of methionine 7 to alanine results in a moderate-affinity heme sensor, HS1-M7A, which exhibits a *K*_D_^Fe(III)^ of 2 μM and *K*_D_^Fe(II)^ of 25 nM (18). Mutation of both methionine 7 and histidine 102 to alanine results in an HS1 variant, HS1-M7A/H102A, that cannot bind heme, *K*_D_^Fe(III)^ of >20 μM and *K*_D_^Fe(II)^ of >20 μM, and serves as a control to determine if there are heme-independent perturbations to sensor fluorescence (18).

In wild-type (WT) M. smegmatis, both HS1 and HS1-M7A exhibit a dose-dependent increase in the eGFP/mKATE2 fluorescence ratio in response to succinylacetone (SA), an inhibitor of the heme biosynthetic enzyme porphobilinogen synthase (Fig. 2A and B) (23, 24). In contrast, HS1-M7A/H102A did not exhibit SA-dependent changes in eGFP/mKATE2 fluorescence ratios (Fig. 2C). For reference, the dose (500 μM) and exposure time (16 to 24 h) of SA utilized in these experiments resulted in an ~5-fold decrease in total cellular heme (Fig. 2D). Together, these data indicated that both HS1 and HS1-M7A were competent for sensing intracellular labile heme in M. smegmatis. By adapting previously established sensor calibration procedures (18) to M. smegmatis (see Materials and Methods), we estimated that the heme occupancies of HS1 and HS1-M7A were >85% and ~50%, respectively, making HS1-M7A ideally suited for measuring labile heme in M. smegmatis (Fig. S1A to C).

In the avirulent M. tuberculosis strain mc^2^6230 (WT M. tuberculosis), the heme sensors HS1 and HS1-M7A likewise exhibited heme-dependent fluorescence responses, while the control, HS1-M7A/H102A, did not (Fig. 3A to C). The *in situ* calibration of the sensors in M. tuberculosis revealed that HS1 is saturated with heme, like in M. smegmatis, and HS1-M7A is <20% bound to heme, unlike in M. smegmatis, where HS1-M7A is ~50% bound to heme (Fig. S1D to F). Labile heme was only completely depleted from HS1 after repeated exposure to 500 μM SA, which was applied every 72 h for 13 days (Fig. 3B). This result is consistent with our findings that it takes between 8 and 16 days of continuous exposure to 500 μM SA to deplete heme by 30% and at least 24 days to deplete heme up to ~50% (Fig. 3D). The relatively muted effect of SA on intracellular heme in M. tuberculosis compared to M. smegmatis may be due to limited uptake of SA and slow growth rates in the former (25). Both exogenous hemin chloride (referred to here as heme) (Fig. 3A and C) and hemoglobin (Hb) (Fig. 3C) significantly increased labile heme as detected by HS1-M7A, which implied that scavenged heme contributed to the labile heme pool. The heme biosynthetic precursor ALA had little effect on the labile heme pool of M. tuberculosis (Fig. 3C), which suggested that heme synthesis and/or partitioning of synthesized heme into the labile heme pool is tightly regulated. The observation that M. smegmatis has a greater concentration of bioavailable heme than M. tuberculosis, as assessed by HS1-M7A heme loading, is not due to higher levels of total heme in M. smegmatis. M. tuberculosis has nearly 7 times more heme per milligram of protein than M. smegmatis (Fig. S1G). Therefore, the reduced labile heme levels of M. tuberculosis result from differences in heme speciation and buffering between M. tuberculosis and M. smegmatis.

### Exogenous versus endogenous heme utilization in M. smegmatis.

**FIG 4:**
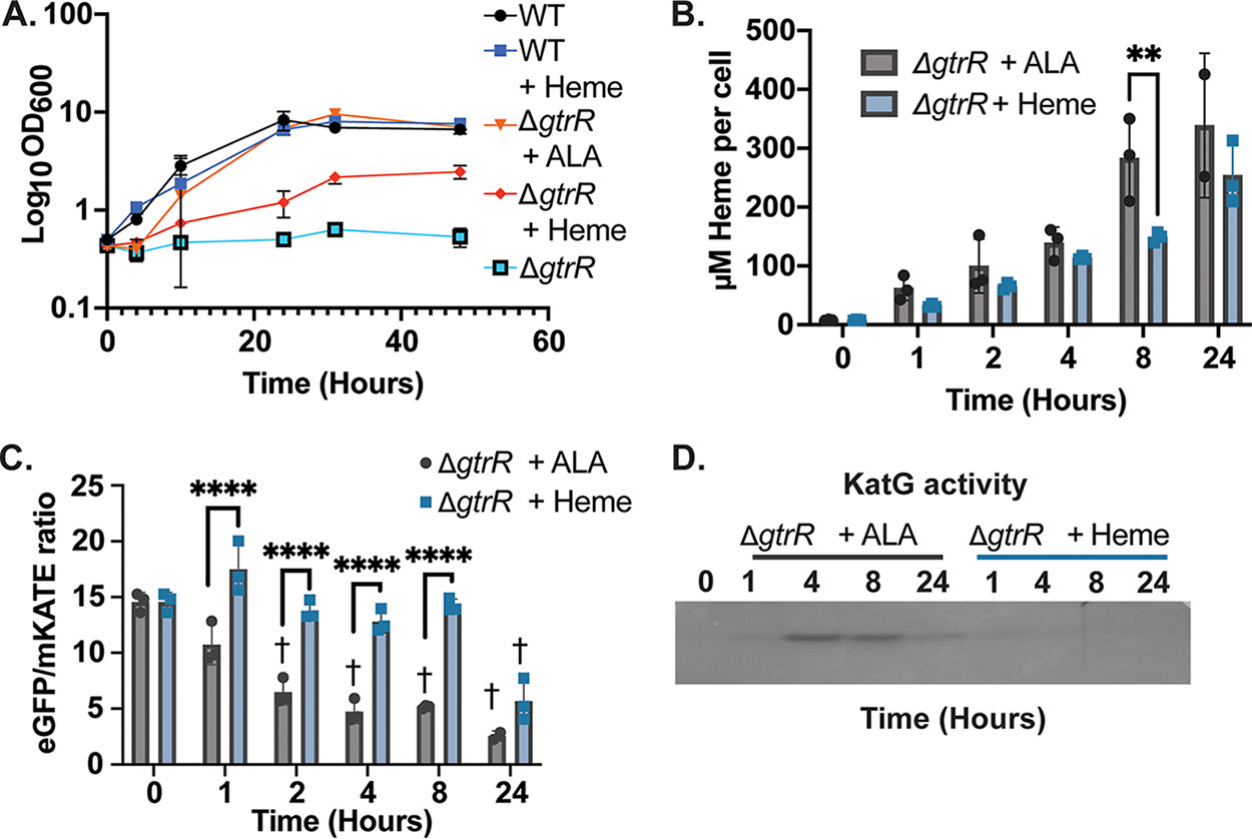
Utilization of exogenous versus endogenous heme in M. smegmatis. (A to D) Effects of ALA (5 μg/mL) or hemin chloride (50 μM) supplementation on (A) growth rate, (B) total intracellular heme, (C) HS1-M7A-detected labile heme, and (D) activity of the heme-dependent catalase-peroxidase KatG in Δ*gtrR* M. smegmatis cells. In panel A, growth curves represent the average optical density of triplicate cultures. In panels B and C, data represent the mean ± SD (error bars) of triplicate cultures. In panel B, the statistical significance was assessed by two-way ANOVA with a Bonferroni *post hoc* test: **, *P = *0.0015. In panel C, the statistical significance was assessed by two-way ANOVA with a Bonferroni *post hoc* test. Black asterisks denote statistically significant differences at each time point: ****, *P* < 0.0001. Gray crosses denote statistically significant differences relative to “time zero” for each set of treatments. †, *P < *0.0001. In all panels, differences that are not statistically significant are unlabeled. The zymogram depicted in panel D is representative of 2 independent trials.

Since mycobacteria can both synthesize and take up heme (10, 26), we sought to determine if there was a difference in the bioavailability and utilization of exogenously imported versus endogenously synthesized heme. Treatment of WT M. smegmatis with the heme synthesis inhibitor SA decreases bioavailable heme (Fig. S2A). However, exogenous heme treatment does not contribute to the bioavailable heme pool as measured by the HS1-M7A heme sensor (Fig. S2B). This led us to consider that M. smegmatis may not utilize exogenous heme as effectively as *de novo*-synthesized heme. To test this hypothesis, we generated a heme auxotrophic strain of M. smegmatis with a deletion of the first heme synthesis enzyme, glutamyl tRNA reductase (GtrR) (10). M. smegmatis Δ*gtrR* exhibited a heme auxotrophy (Fig. 4A, black squares with cyan shading), and addition of 5 μg/mL ALA, which restored heme synthesis (Fig. 4B, gray bars), rescued growth to that of WT M. smegmatis (Fig. 4A, orange triangles and black circles, respectively). However, M. smegmatis Δ*gtrR* treated with 50 μM exogenous heme exhibited a substantial growth defect (Fig. 4A, red diamonds) compared to WT M. smegmatis (Fig. 4A, black circles) and M. smegmatis Δ*gtrR* grown with ALA (Fig. 4A, orange triangles). This growth defect was not due to heme toxicity, as WT M. smegmatis grown with 50 μM hemin chloride (Fig. 4A, blue squares) had growth rates similar to those of untreated cells (Fig. 4A, black circles). These results suggested that M. smegmatis does not utilize imported heme as efficiently as endogenously synthesized heme.

To determine if the growth disparity between endogenous and exogenous heme utilization corresponded to differences in intracellular heme accumulation and/or bioavailability, we measured total and labile heme in M. smegmatis Δ*gtrR* grown in the presence of heme or ALA. M. smegmatis Δ*gtrR* cells were starved of heme and ALA for 18 h, which was sufficient to reduce total heme (Fig. 4B) and deplete labile heme (Fig. 4C). M. smegmatis Δ*gtrR* was then supplied with either 5 μg/mL ALA to initiate endogenous heme synthesis or 50 μM heme. Addition of both ALA and heme increased total heme to a similar level in M. smegmatis Δ*gtrR* over 24 h (Fig. 4B). However, ALA more rapidly increased the bioavailable labile heme pool than imported heme over the same time span (Fig. 4C). Addition of ALA resulted in a significant increase in labile heme within 2 h, whereas it took more than 8 h for exogenous heme to have the same effect. These results imply that the poor rescue of M. smegmatis Δ*gtrR* by exogenous heme is caused by the slow population of the labile heme pool compared to ALA.

The heme-dependent catalase-peroxidase KatG, which is both cytosolic and secreted (27), plays an important role in detoxifying reactive oxygen species in mycobacteria and is important for mycobacterial virulence (28). To determine if the difference in utilization of exogenous versus biosynthesized heme affected the activity of this hemoprotein, we measured KatG activity in M. smegmatis Δ*gtrR* using an in-gel catalase-peroxidase activity assay (29, 30). Upon depletion of heme, M. smegmatis Δ*gtrR* did not have active KatG (Fig. 4D, time zero). Growth of M. smegmatis Δ*gtrR* with ALA yielded active KatG within 4 h (Fig. 4D). However, addition of exogenous heme did not yield active KatG even after 24 h (Fig. 4D). Thus, synthesized heme rescues KatG activity of M. smegmatis Δ*gtrR*, in contrast to imported heme. Altogether, these results revealed that M. smegmatis Δ*gtrR* utilizes imported heme to support growth and metabolism, but it is less bioavailable than endogenously synthesized heme.

### Heme uptake and utilization in M. tuberculosis.

**FIG 5:**
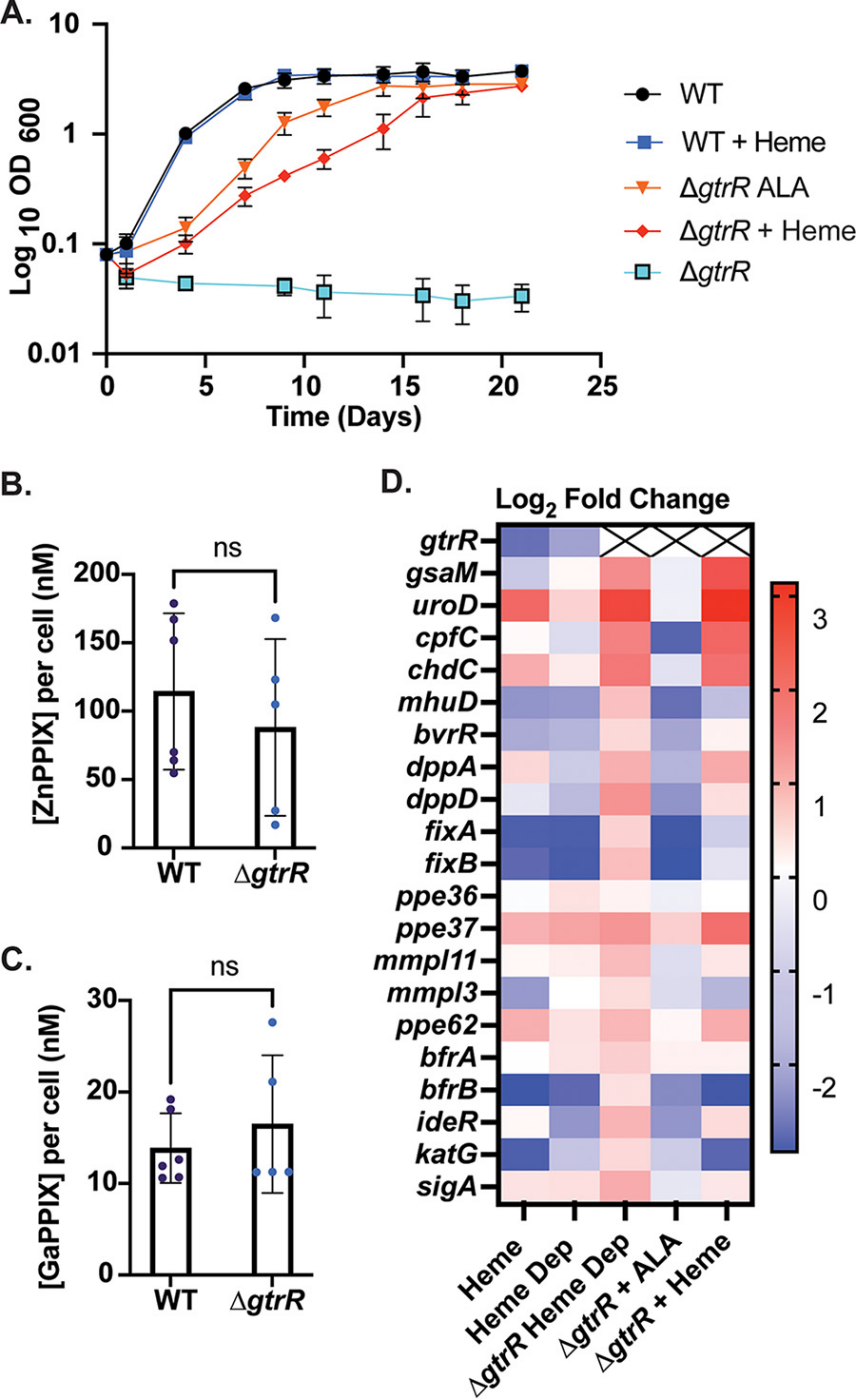
Uptake and utilization of exogenous heme by M. tuberculosis. (A) Effects of ALA (5 μg/mL) or hemin chloride (25 μM) supplementation on growth of WT or Δ*gtrR* M. tuberculosis strains. (B and C) Whole-cell fluorescence of WT and Δ*gtrR* M. tuberculosis cells treated with 1 μM the fluorescent heme analogs ZnPPIX and GaPPIX, respectively, for 48 h. (D) Heat map of log_2_ fold change of M. tuberculosis cultures compared to WT M. tuberculosis cultured in 7H9+ADS. The data shown are averages of two biological replicates. Xs represent the log_2_ fold change of <–6 for genetic knockouts. In panel A, growth curves represent the average cell density of triplicate cultures. In panels B and C, the statistical significance was assessed by a two-tailed unpaired Student’s *t* test; *P* = 0.4908 and 0.4693, respectively.

To examine whether exogenous and *de novo*-synthesized heme are also utilized differently in M. tuberculosis, we generated heme auxotrophic M. tuberculosis strains by deletion of the *gtrR* gene and determined their ability to utilize exogenous heme. As expected, M. tuberculosis *ΔgtrR* exhibited heme auxotrophy (Fig. 5A, cyan squares). As observed for M. smegmatis, ALA rescued M. tuberculosis *ΔgtrR* cells more efficiently than exogenous heme (Fig. 5A, orange triangles and red diamonds, respectively) However, addition of ALA to M. tuberculosis *ΔgtrR* (Fig. 5A, orange triangles) did not restore WT growth, in contrast to M. smegmatis (Fig. 4A, black circles). These results suggest that import of ALA under these conditions is not sufficient to fully support growth of M. tuberculosis *ΔgtrR*.

To determine if ablation of *gtrR* disrupted the uptake of exogenous heme, we measured the accumulation of the fluorescent heme analogs zinc(II) protoporphyrin IX (ZnPP) and gallium(III) protoporphyrin IX (GaPP) in WT M. tuberculosis and M. tuberculosis Δ*gtrR* via whole-cell fluorescence. The lack of a statistically significant difference in ZnPP and GaPP accumulation between WT M. tuberculosis and M. tuberculosis Δ*gtrR* (Fig. 5B and C) implied that the defect in exogenous heme bioavailability is not due to a defect in heme import in M. tuberculosis Δ*gtrR* cells.

### Heme-dependent transcriptional responses in M. tuberculosis.

In order to assess the status of heme metabolism and utilization between *de novo*-synthesized and imported heme we examined the expression of a wide panel of heme homeostasis genes in response to heme in WT M. tuberculosis and M. tuberculosis Δ*gtrR* cells using reverse transcriptase quantitative PCR (RT-qPCR) (Fig. 5D). Markers for heme metabolism included transcripts of genes encoding the heme biosynthetic enzymes GtrR, GsaM, CpfC, Urod, and ChdC (10), the heme-degrading enzyme MhuD (31), heme acquisition and import factors, e.g., Ppe36, Ppe37, Ppe62, and DppA (7, 12, 17), a previously suspected heme transporter, MmpL3 (13), iron storage proteins, BfrA and BfrB (32), a heme-dependent peroxidase, KatG (33), and an iron homeostatic factor, IdeR (34). WT M. tuberculosis and M. tuberculosis Δ*gtrR* cells were cultured in 7H9 broth supplemented with albumin, dextrose, and salt (7H9+ADS, see Materials and Methods) with the addition of 25 μM heme until growth reached saturation. Cells were then either washed and diluted 1:5 into 7H9+ADS without heme for 3 days (heme depleted) or diluted 1:5 into 7H9+ADS with 25 μM heme for 3 days (plus heme). M. tuberculosis Δ*gtrR* plus ALA cells were cultured in 7H9+ADS with 5 μg/mL ALA to saturation and then diluted 1:5 in medium with 5 μg/mL ALA for 3 days. As a control, untreated WT M. tuberculosis cells were cultured then diluted 1:5 in 7H9+ADS for 3 days and served as a reference to evaluate heme status in all mutants and growth conditions.

In WT M. tuberculosis, exogenous heme regulates the expression of the early and late enzymes of the heme biosynthetic pathway distinctly, with heme causing a 4-fold decrease in the *gtrR* transcript and a 2.5-fold increase in the *uroD* and *chdC* transcripts (Fig. 5D and Data set 1). The results suggest that genes encoding early and late heme synthetic enzymes are differentially regulated. Additionally, heme treatment of WT M. tuberculosis upregulated only the cell surface heme transport genes *ppe37* and *ppe62*, while other heme uptake genes did not have significant increases in transcript. Exogenous heme downregulated the transcription of *mhuD* and *bfrB*, the heme-degrading heme oxygenase and the ferritin, respectively, indicating that heme degradation to release iron may not occur under iron-replete growth conditions. Of note, WT M. tuberculosis cells depleted of exogenous heme showed lingering effects of heme exposure in their transcriptional profiles (Fig. 5D, column 2). This may be due to the continued internalization of recalcitrant heme retained at the cell surface even after washing and removal of exogenous heme from the cells and culture media.

Heme-depleted M. tuberculosis *ΔgtrR* exhibited a transcriptional profile consistent with heme starvation, with all transcripts encoding heme biosynthetic and uptake proteins being induced (Fig. 5D). The addition of ALA to M. tuberculosis *ΔgtrR*, but not exogenous heme, generated a heme-replete state, with transcription of heme synthesis and transport genes being repressed or restored relative to the WT (Fig. 5D). The differential transcriptional responses of M. tuberculosis *ΔgtrR* to heme versus ALA addition are consistent with endogenously synthesized heme being more bioavailable than exogenously supplied heme (Fig. 5D and Fig. S3A). Interestingly, restoring heme synthesis in M. tuberculosis *ΔgtrR* with ALA resulted in iron limitation, as inferred from the ~3-fold decrease in the transcription of the iron homeostatic genes *ideR* and *bfrB*, which positively correlate with iron levels (32, 35). The increased *ideR* and *bfrB* transcription in heme-depleted M. tuberculosis *ΔgtrR*, which cannot synthesize heme, is consistent with the notion that heme synthesis limits intracellular iron availability. Although exogenous heme does not rescue the heme deficiency of M. tuberculosis *ΔgtrR*, it does repress *bfrB*, indicating that exogenous heme alters iron homeostasis in M. tuberculosis Δ*gtrR* (Fig. 5D and Fig. S3B), albeit through an unknown mechanism. Surprisingly, *mhuD* transcription did not correlate with heme levels as expected, with heme-depleted M. tuberculosis *ΔgtrR* exhibiting elevated *mhuD* transcripts, and M. tuberculosis *ΔgtrR* grown with either ALA or heme exhibiting reduced *mhuD* transcripts. Altogether, transcriptomic profiling suggests that ALA, but not exogenous heme, effectively alleviates the heme deficiency of *ΔgtrR* cells and that heme synthesis, and exogenous heme to a certain degree, contributes to limiting cellular iron.

### Exogenous heme decreases porphyrin synthesis in M. tuberculosis.

Given that heme treatment reduces the transcription of genes encoding early heme biosynthetic enzymes (Fig. 5D), we predicted that heme feedback inhibition may regulate heme synthesis in mycobacteria, as has been found to occur in other bacteria (10). To determine if exogenous heme inhibits heme synthesis in mycobacteria, we measured fluorescent porphyrins as an indicator of flux through the heme biosynthetic pathway. Porphyrins fluoresce at ~650 nm when excited with 400 nm light. The levels of these species, which we refer to as “free porphyrins” (FPs), would include the heme biosynthetic intermediate coproporphyrin along with any porphyrinogens that oxidize in air upon cell lysis. An increase in FPs would imply that there is either a block in late stages of heme synthesis or that iron insertion by ferrochelatase does not keep up with increased flux through the heme synthetic pathway.

We found that WT M. tuberculosis lysates consistently exhibited porphyrin fluorescence emission in standard culture conditions. When these cells were treated with the heme synthetic inhibitor SA, FP emission decreased, consistent with the SA-mediated inhibition of porphobilinogen synthase, an early step of heme synthesis (Fig. S4A and B). Most notably, heme addition to WT M. tuberculosis decreased FP emission to levels similar to those measured with addition of SA, suggesting that heme acted as a feedback inhibitor of heme synthesis (Fig. S4A and B). As expected, FP levels in WT M. tuberculosis were increased by addition of ALA (Fig. S4C). Interestingly, heme-dependent inhibition of FP accumulation was not observed in WT M. smegmatis (Fig. S4D). The assignment of the 650-nm-emitting species as porphyrin was validated by the loss of fluorescence emission in the FP spectral window in heme-deficient Δ*gtrR* mutants of M. tuberculosis (Fig. S4E) and M. smegmatis (Fig. S4F). Taken together with the reduced transcription of the genes encoding the initial heme biosynthesis enzymes in WT M. tuberculosis when treated with heme (see previous section), our data suggest that exogenous heme inhibits heme biosynthesis in M. tuberculosis.

### Role of endogenous heme synthesis in macrophage infection.

**FIG 6:**
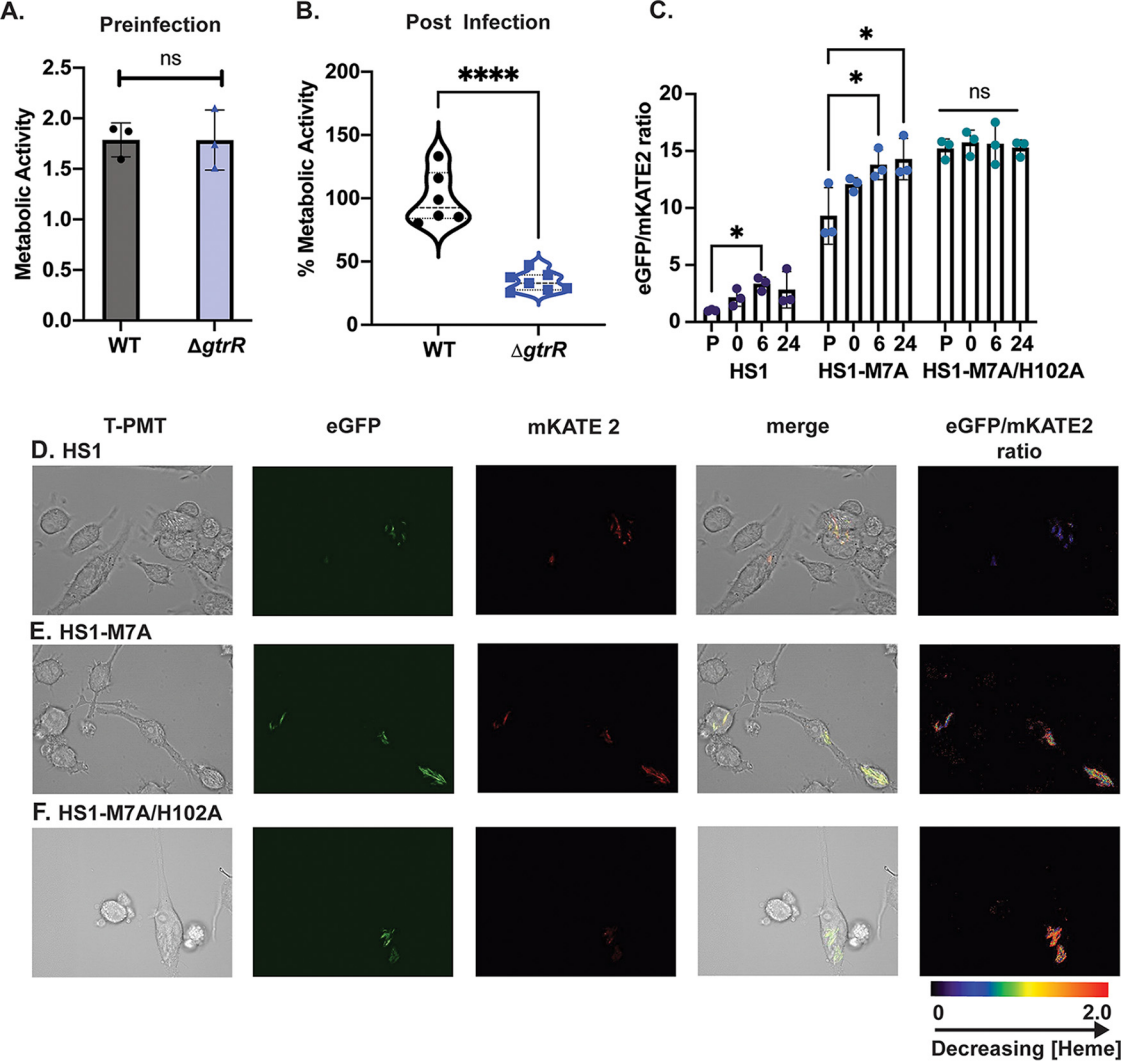
Role of heme synthesis in a macrophage infection model. (A) Formazan absorbance as turnover of MTT in WT M. tuberculosis and Δ*gtrR* M. tuberculosis of 2 × 10^6^ cells prior to macrophage infection; 2 × 10^6^ cells are equivalent to 100% engulfment and survival in macrophage infection used for panel B. (B) Formazan absorbance reported as % metabolic activity of WT M. Tuberculosis and Δ*gtrR* M. Tuberculosis isolated from RAW 246.7 macrophages after 24 h of infection from 2 independent trials. WT M. tuberculosis, *n* = 6; ΔGtrR M. tuberculosis, *n* = 7. (C) Fluorescence ratio of WT M. tuberculosis expressing HS1, HS1-M7A, and HS1-M7A/H102A. Preinfection (P), 0, 6, and 24 are readings in intact macrophages at 0, 6, and 24 h, respectively, after infection and washing of extracellular M. tuberculosis (see Materials and Methods). (D–F) Confocal microscopy of M. tuberculosis engulfed via macrophages corresponding to the 24-h time point in panel C. In panel A, the data represent the mean ± SD (error bars) of triplicate cultures. The statistical significance was assessed by an unpaired two-tailed Student’s *t* test. ns, Nonsignificant *P > *0.9930. In panel B, statistical significance was assessed by an unpaired two-tailed Student’s *t* test; *P* < 0.0001. In panel C, statistical significance was calculated via a one-way ANOVA with a Dunnett’s multiple comparison test for each sensor data set using the preinfection (P) time point as a control. *, *P* = 0.0386 for HS1; *, *P* = 0.0297 and 0.0178 for the HS1-M7A 6- and 24-h time points, respectively. For HS1-M7A/H102A all *P* ≥ 0.9087.

Based on the importance of heme biosynthesis for growth of M. tuberculosis, we sought to examine whether heme synthesis is also important for survival and replication of M. tuberculosis in macrophages. Toward this end, we employed an *in vitro* assay of intracellular infection using RAW 264.7 macrophages in order to determine differences in metabolic activity between Δ*gtrR* and WT M. tuberculosis in macrophages (36). We measured the reduction of the tetrazolium dye MTT [3-(4,5-dimethyl-2-thiazolyl)-2,5-diphenyl-2H-tetrazolium bromide] to a purple formazan as a readout of metabolic activity and viability in M. tuberculosis and M. smegmatis (37, 38). After heme depletion, the MTT-reducing activities of WT M. tuberculosis and M. tuberculosis Δ*gtrR* were similar (Fig. 6A). However, the metabolic activity of M. tuberculosis Δ*gtrR* cells isolated from RAW macrophages 24 h after infection was ~50% decreased compared to that of WT M. tuberculosis (Fig. 6B). A similar phenotype was observed for M. smegmatis 4 h after infection (Fig. S5). These results highlight the importance of *de novo* heme synthesis for survival of mycobacteria during macrophage infection.

To determine if the requirement for heme synthesis in mycobacteria to survive macrophage assault correlated with changes in labile heme, we infected RAW macrophages with WT M. tuberculosis expressing the labile heme sensors. The heme sensor ratio was measured in WT M. tuberculosis within intact macrophages by fluorescence spectroscopy of the whole-cell population (Fig. 6C) and by fluorescence microscopy (Fig. 6D to F). Labile heme levels were significantly reduced in cells after infection compared to preinfection levels as measured by the HS1-M7A sensor, with little variation in HS1-M7A/H102A, which does not bind heme (Fig. 6C, blue circles and green circles respectively). Fluorescence microscopy confirmed that WT M. tuberculosis expressing the labile heme sensors within macrophages had expected ratios for the HS1, HS1-M7A, and HS1-M7A/H102A sensor variants (Fig. 6D to F). In total, these data show that during macrophage infection, bioavailable heme in M. tuberculosis decreases, suggesting that heme synthesis is required for survival and/or replication of M. tuberculosis in macrophages.

## DISCUSSION

The importance of heme in M. tuberculosis physiology is underscored by the availability of at least two independent routes for heme acquisition in addition to *de novo* heme synthesis in M. tuberculosis (12, 39). Heme-dependent proteins, including KatG (40, 41), cytochrome P450s (42), and the Dos two-component regulatory system (43), are required for the survival and virulence of M. tuberculosis in infection models (28, 40). The necessity of heme for M. tuberculosis during infection has led to the proposal of targeting both heme uptake and heme synthesis pathways of M. tuberculosis for potential anti-M. tuberculosis therapies (6, 9, 12). However, the relative contributions of *de novo*-synthesized and exogenously scavenged heme toward labile bioactive heme pools and protein hemylation were unknown.

In this work, we deployed heme sensors to measure the labile heme pool in both M. smegmatis and M. tuberculosis. We have shown that M. smegmatis and M. tuberculosis have different levels of total heme and labile heme per cell and that labile heme is a good indicator of the bioactivity of subcellular heme pools in mycobacteria, including for activation of KatG, which is necessary for survival of M. tuberculosis in the macrophage (28, 40) and is expressed in clinical isolates of TB patients (41). Additionally, we found that relative to endogenously synthesized heme, imported heme was poorly utilized by mycobacteria and was inefficient at (i) rescuing the growth of a heme-deficient Δ*gtrR* strain (Fig. 4A and 5A), (ii) reversing the transcriptional markers of heme deficiency in Δ*gtrR* cells (Fig. 5D), (iii) contributing to the labile heme pool (Fig. 4C), and (iv) activating the heme enzyme KatG (Fig. 4D).

One potential explanation for the apparent poor bioavailability of exogenously supplied heme is that it is rapidly degraded. However, we counterintuitively found that the transcript for the heme-degrading heme oxygenase, MhuD, is repressed by addition of exogenous heme and induced under heme-deficient conditions (Fig. 5D), suggesting that the differences in bioavailability between exogenously transported and endogenously made heme are not caused by heme degradation. Nonetheless, we cannot rule out this possibility, as we have been unable to directly measure mycobilin, a product of heme degradation in mycobacteria (31), from cell extracts and therefore cannot directly determine heme degradation in M. tuberculosis.

An alternative explanation for the difference in exogenous and endogenous heme bioavailability is that M. tuberculosis may traffic heme differently depending upon whether it is synthesized or imported. This could be due to oxidation state differences between synthesized and imported heme, which may subsequently alter how heme is trafficked within the cell. It is expected that the heme iron is in the oxidized Fe^3+^ state when bound to ChdC, as coproheme decarboxylase activity requires ferric iron in its resting state (44, 45). In contrast, exogenous heme may be reduced to the ferrous Fe^2+^ state during transport as shown for Staphylococcus aureus, where the heme-binding ABC transporter protein IsdE is selective for ferrous over ferric heme (46). Another possibility is that exogenous heme is sequestered into microcompartments or lipid membranes. In mammalian cells and Caenorhabditis elegans, transient absorption microscopy of live cells and animals, respectively, identified heme granules upon exposure to exogenous heme sources (47). In addition, the fission yeast Schizosaccharomyces pombe and human erythroid cells were found to traffic exogenous heme via endocytic vesicles (48, 49). Similarly, M. tuberculosis has multiple heme uptake pathways (7, 12) and uses lipid vesicles as a means to adapt to stress (50, 51). However, the mechanisms that M. tuberculosis uses to differentially handle exogenous and endogenous heme remain to be determined. It is important to note that the relative preferences for *de novo*-synthesized heme versus exogenously scavenged heme may depend on environmental circumstances, including alterations in iron or oxygen availability or response to the host immune system.

Using an M. tuberculosis-macrophage infection assay, we found that survival of the heme synthetic mutant, Δ*gtrR*, was impaired in infected macrophages compared to WT M. tuberculosis (Fig. 6B) and M. smegmatis (Fig. S5). This may be due to the ability of macrophages to induce heme limitation in M. tuberculosis over the first 24 h of infection (Fig. 6C). Our findings that *de novo*-synthesized heme is important for M. tuberculosis survival is also supported by prior studies indicating that many enzymes of the M. tuberculosis heme biosynthetic pathway were upregulated in TB patient lungs compared with *in vitro* growth conditions (52) and that the granuloma contains many host heme-scavenging proteins, including hemopexin and haptoglobin, signifying that the host restricts heme availability at the site of infection (53). Future work will involve assessing the role of M. tuberculosis heme biosynthetic enzymes and heme homeostasis in nonattenuated strains in mouse and macrophage models of M. tuberculosis infection.

To balance the metabolic need for heme while also mitigating heme toxicity, many pathogens have the capacity to regulate heme synthesis in response to changes in cellular heme concentrations and/or heme acquisition (54–58). For instance, in Staphylococcus aureus, heme acquisition and synthesis are mechanistically linked via the ability of the heme oxygenase IsdG to bind and inhibit ferrochelatase (54). In addition, the heme regulatory factor, HemX, posttranslationally upregulates GtrR levels in response to heme deficiency (55). Our results in M. tuberculosis suggest that heme acts as a feedback inhibitor by repressing transcription of genes encoding the early heme synthetic enzymes GtrR and GsaM (Fig. 5D), which produce the first committed metabolite for heme synthesis, ALA (Fig. 1). Conversely, heme deficiency, as shown in Δ*gtrR* cells, results in the upregulation of transcripts for heme synthetic enzymes. However, the molecular details of these regulatory mechanisms in mycobacteria are poorly understood and require further study.

A surprising finding of our work was the significant quantities of free porphyrins in WT M. tuberculosis (Fig. S4). The buildup of heme intermediates is considered disadvantageous due to the inherent cytotoxicity of porphyrins (59). For example, in M. smegmatis, the buildup of porphyrins in cultures during the transition to dormancy increases the susceptibility of M. smegmatis to photoinactivation (60). The high free porphyrin levels in M. tuberculosis might provide a basis for therapies using light-enhanced porphyrin toxicity, as has been shown for other bacteria (61) and in cancers (62).

Our study is the first to demonstrate that heme synthesized *de novo* is more bioavailable to mycobacteria than imported heme and that *de novo* heme synthesis is important for M. tuberculosis survival during macrophage infection. These findings bolster the case for targeting heme synthesis in new antimycobacterial therapeutics. Future studies will aim at understanding the mechanisms of how M. tuberculosis coordinates heme uptake and synthesis and determining how M. tuberculosis distinguishes between different heme sources.

## MATERIALS AND METHODS

### Bacterial strains and reagents.

M. smegmatis mc^2^155 and M. tuberculosis mc^2^6230 (H37Rv ΔRD1 ΔpanCD) were obtained from laboratory stocks. For knockouts and transformations, the strains were grown in Middlebrook 7H9 broth (Difco, Sparks, MD) supplemented with 10% (vol/vol) oleic acid-albumin-dextrose catalase (OADC; Difco), 0.2% (vol/vol) glycerol, and 0.05% (vol/vol) Tween 80 at 37°C with shaking. Middlebrook 7H10 agar (Difco) supplemented with 10% (vol/vol) OADC and 0.2% (vol/vol) glycerol was used as the solid medium. The following supplements were used at the following concentrations: l-pantothenate, 50 μg/mL; heme and ALA, as indicated. The plasmid pYUB1471, shuttle phasmid phAE159, and phage phAE280 were obtained from laboratory stocks (63). Hygromycin (Gold Biotechnology, St. Louis, MO) was used at concentrations of 50 μg/mL for mycobacteria and 150 μg/mL for Escherichia coli. All the supplements were obtained from Sigma-Aldrich or Thermo (Fisher) Scientific. All primers used for generation of mutant and sensor strains are listed in Table S1.

### Generation of Δ*gtrR* mutants.

The genes for *gtrR* were identified by bioinformatics analysis in M. smegmatis and M. tuberculosis. The deletion mutants of *gtrR* were generated by specialized transduction as described previously (63). The transductants were selected on plates containing hygromycin as the selective marker and ALA. The hygromycin cassette was excised from the knockout strains using the phage phAE280 and sucrose selection (63). The deletion and unmarked strains were confirmed by PCR and sequencing. The primers used to generate and confirm the mutant strains are listed in supplementary material.

### Generation of heme sensors for mycobacteria.

The heme sensors HS1, HS1-M7A, and HS1-M7A/H102A were cloned under the control of the G13 promoter in an episomal mycobacterial plasmid (18, 64, 65). The resulting plasmids expressing HS1 (pYUB1872), HS1-M7A (pYUB1874), and HS1-M7A/H102A (pYUB1876) were confirmed by restriction digestion and sequencing. Plasmids were electroporated in various strains of mycobacteria using the following settings (2.5 kV, 25 mF, and 1,000 Ω) (66). Transformants were selected on 7H10 agar plates containing the necessary supplements and kanamycin (40 μg/mL). Plates were incubated at 37°C for 3 days for M. smegmatis and 4 to 6 weeks for M. tuberculosis.

### M. smegmatis growth and heme depletion.

M. smegmatis strains were grown with shaking at 170 to 200 rpm at 37°C in Middlebrook 7H9 medium (Difco) supplemented with 10% ADS (albumin, dextrose and salt) and 0.05% Tween 80. ADS was composed of 5% bovine serum albumin (BSA; Fraction V Gemini Biosciences), 2% glucose, and 0.85% sodium chloride in water. Sensor strains were grown with 40 μg/mL kanamycin sulfate. Δ*gtrR* strains were supplemented with either 1.5 μg/mL ALA or 25 μM hemin chloride. For depletion, cells were grown in ALA or heme supplemented medium to an optical density at 600 nm (OD_600_) of ~1. Cells were pelleted and washed 3 times with sterile water before resuspension at 1 OD_600_ in 7H9 medium with no ALA or heme. Cells were grown in depleted medium for 18 h. After depletion, the cells were resuspended at 1 OD_600_, and measurements were taken as heme depleted the cells. The cells were then supplemented with 5 μg/mL ALA or 50 μM hemin chloride for ALA and heme studies.

### M. tuberculosis growth and heme depletion.

M. tuberculosis cells were grown shaking at 170 to 200 rpm at 37°C in Middlebrook 7H9 medium (Difco) supplemented with 10% ADS, 0.2% Casamino Acids (Difco), 0.02% tyloxapol, and 50 μg/mL calcium pantothenate (Sigma). Cells were grown in 10- to 11-mL cultures in closed 30-mL square polyethylene terephthalate copolyester, glycol modified (PETG) bottles (Fisher). Δ*gtrR* M. tuberculosis strains were grown with 5 μg/mL ALA and/or 25 μM hemin chloride. For heme depletion, Δ*gtrR* cells were pelleted, washed 1 time in 7H9 medium, and resuspended in 7H9 medium without hemin or ALA supplementation. Sensor strains were grown with 40 μg/mL kanamycin.

### Labile heme assay.

Cells at each time point were taken, and the OD_600_ was measured to determine cell density. Cells were pelleted and washed in water 2 times before being suspended in phosphate-buffered saline (PBS) at an OD_600_ of 1 for M. tuberculosis and of 10 for M. smegmatis for sensor fluorescence measurements. For sensor fluorescence, 200 μL of cells was plated in 96-well flat bottom Greiner FLUOROTRAC plates as technical duplicates. WT M. smegmatis and M. tuberculosis cells not expressing sensor were measured and subtracted as background fluorescence for both the eGFP and mKATE2 channels. Sensor fluorescence was measured on a BioTek Synergy MX plate reader, with an excitation at 480 nm and emission at 510 nm for eGFP and an excitation at 580 nm and emission at 620 nm for mKATE2; slit widths for excitation and emission were 9 nm. Multiple reads over 5 to 10 min were taken to account for variability in fluorescence over time. Multiple reads and technical replicates were averaged as one ratio. The reported mean and standard deviation were calculated from biological replicates.

### *In situ* heme sensor calibration.

In order to relate the sensor fluorescence ratio to the fractional heme occupancy of the sensor, a previously established method to calibrate the heme sensors in yeast was adapted for mycobacteria (18).

(1) $$\mathrm{\% bound}=\left(\frac{R_{\mathrm{expt}}\;-\;R_{\min}}{R_{\max}\;-\;R_{\min}}\;\right)\;\times \;100$$

The amount of heme bound to the sensor, % bound, can be quantified by determining the sensor eGFP/mKATE2 fluorescence ratio under any given experimental condition, *R*_expt_, relative to the eGFP/mKATE2 fluorescence ratios when the sensor is 0% (*R*_min_) and 100% (*R*_max_) bound to heme, respectively. The theoretical limit for *R*_max_ is ~0 due to the >99% efficiency of energy transfer between GFP and heme. This was confirmed by permeabilizing cells and adding 50 μM hemin chloride, which saturated HS1 and gave a ratio approaching 0 (Fig. S1A and D). *R*_min_ was determined by growing parallel cultures of cells expressing HS1-M7A/H102A, which cannot bind heme (Fig. S1C and F).

To calibrate the sensors, cells were washed with water and resuspended in PBS at an OD_600_ of 10, and prepermeabilization fluorescence was measured as detailed for the labile heme assay described above. Cells were resuspended in a previously reported permeabilization buffer used for terminal deoxynucleotidyl transferase dUTP nick end labeling (TUNEL) assays in M. tuberculosis (67). The permeabilization buffer of 0.1% Triton X-100 in 0.1% sodium citrate was supplemented with 1 mM ascorbate. To saturate the sensor, cells were treated with and without 50 μM hemin chloride for 30 min with shaking at 37°C. Cells were pelleted, washed 2 times with PBS, and resuspended in 1 mM ascorbate in PBS, and sensor fluorescence was measured and reported as permeabilized (no heme) and saturated (plus heme).

### Total heme assay.

Total heme measurements were based on a previously reported porphyrin fluorescence assay (68). Briefly, M. smegmatis or M. tuberculosis cells were taken at time points and washed as for labile heme measurements described above. After suspension at an OD_600_ of 10 in 1 mL PBS for M. smegmatis or ~1 OD_600_ in 1 mL PBS for M. tuberculosis, 500 μL of cells was pelleted and frozen at −80°C. Cells pellets were resuspended in 500 μL of 20 mM oxalic acid and allowed to sit at 4°C overnight. To each cell suspension, 500 μL of 2M oxalic acid was added. Cells were mixed and divided into two 500-μL aliquots. One was stored at in the dark at room temperature as a blank. The other was boiled at 100°C for 30 min covered. Blanks and boiled samples were centrifuged at 21,100 × *g* for 2 min to remove cell debris. For fluorescence measurements, 200 μL of supernatant was plated as technical duplicates in 96-well black flat-bottom Greiner FLUOROTRAC plates. Fluorescence was measured on a Tecan Infinite 200 Pro plate reader with excitation at 400 nm and emission at 608 nm and 662 nm. Emission spectra from 600 to 700 nm were also recorded. Heme concentration was calculated from fluorescence by comparison with heme standards from 1 nM to 500 nM treated with oxalic acid as described above for the cell samples.

### Free porphyrin assay.

Free porphyrin measurements were based on a previously reported assay (68). Briefly, M. smegmatis and M. tuberculosis cells from the total heme assay were used. As in the total heme assay, cells were treated with 20 mM oxalic acid overnight. 2 M oxalic acid was added to samples, and they were allowed to sit for 30 min in the dark. Free porphyrin was measured as fluorescence on a Tecan Infinite 200 Pro plate reader with excitation at 400 nm and emission from 600 to 700 nm. Cells are measured at equivalent OD_600_s, and values are reported in arbitrary fluorescence units (AFU).

### Catalase-peroxidase activity gel.

Catalase-peroxidase activity gels were based on a previously reported assay (29, 30). Briefly, cells were washed as for the labile and total heme measurements. After resuspension at an OD_600_ of 10 in 1 mL PBS, cells were pelleted, and frozen at −80°C. Cells were resuspend in lysis buffer (PBS plus 0.1% Triton X-100, 1 mM EDTA, and 1× protease arrest) and lysed with 0.5 mm zirconium oxide beads in a bullet blender tissue homogenizer at setting 8 for 3 min at 4°C. Cell debris and beads were removed by centrifugation at 21,100 × *g* for 5 min. Lysate was measured for total protein content via the Bradford assay or absorbance at 280 nm. Even loading of total protein was verified by SDS-PAGE. For catalase gels, lysates were separated on a 14% native Novex precast Tris-glycine gel at 4°C for 16 to 18 h. The catalase gel was washed in ultrapure water three times for 15 min each and then incubated with 0.3% H_2_O_2_ for 10 min. The gel was rinsed and added to a mixture of equivalent volumes (~30 mL total) of 2% potassium ferricyanide and 2% iron chloride to stain the gel. The gel was rocked by hand until a faint green color started to appear in the gel, and then the gel was transferred to water and imaged. Catalase bands appeared clear or yellow on the green background of the gel. Gel images were converted to grayscale and inverted for easier viewing in print.

### RT-qPCR.

Cultures (10 mL) of WT M. tuberculosis and Δ*gtrR* M. tuberculosis were grown in duplicate under various test conditions. The cultures were centrifuged, and pelleted cells were resuspended in 1 mL RNA Later stabilization solution overnight at 4°C before storage at −80°C. For RNA extraction, the cells were centrifuged and resuspended in 1 mL of TRIzol reagent (Ambion). The samples were transferred to 2-mL screwcap tubes containing 0.5 mm Zirconia beads and processed in a PowerLyzer 24 homogenizer (Qiagen) for 45 s at 3,500 rpm (×4). The debris was spun down, and the supernatant (~750 μL) was transferred to a fresh tube. An equal volume of absolute ethanol was added and applied to a Zymo-Spin IIICG column. The RNA was extracted by following the Direct-zol RNA MiniPrep Plus protocol, which includes an on-column DNase digestion step. RNA yields were determined using a Qubit RNA BR assay (Invitrogen). cDNA was synthesized using ~400 ng RNA and LunaScript RT supermix (New England Biolabs [NEB]) in a 25-μL reaction using the following program: 2 min at 25°C, 20 min at 55°C, 1 min at 95°C. A No-RT control mix (NEB) was used to eliminate DNA contamination in samples. The cDNA products were quantified in triplicate by real-time PCR, using Luna universal qPCR mastermix (NEB) in a 20-μL reaction and 0.25-μM primer concentrations and running the following program on an ABI 7500 fast real-time system: 2 min at 95°C, 45 cycles of 15 s at 95°C, 30 s at 60°C (plus plate read; SYBR), followed by a dissociation stage of 15 s at 95°C, 15 s at 60°C, and 15 s at 95°C to check the specificity of the products. Threshold cycles were normalized to those for 16S rRNA. Representative samples were also run on a 1.5% agarose gel. All primers used for qPCR are listed in Table S2.

### GaPP and ZnPP uptake.

Gallium(III) protoporphyrin IX (GaPP) and zinc(II) protoporphyrin IX (ZnPP) were both purchased from Frontier Scientific. GaPP and ZnPP stocks were made by dissolving GaPP and ZnPP in dimethyl sulfoxide (DMSO). Concentrations were measured by extinction coefficients reported in the literature (69). GaPP and ZnPP at 1 μM or an equivalent volume of DMSO as the control, were added for the times shown or 48 h in medium (7H9+ADS). Cells were placed at the same OD and washed 2 times in 2% BSA in PBS and 2 times in PBS to remove unbound GaPP or ZnPP. Fluorescence spectra of cells were measured with an excitation of 410 nm (9-nm bandwidth), and emission spectra were read from 500 to 700 nm in 1-nm steps, with peak emission at 585 nm read for GaPP and 589 nm for ZnPP. Fluorescence in DMSO-treated samples was subtracted, and concentration per cell was calculated from a standard curve to account for different quantum yields for GaPP and ZnPP.

### Macrophage infection assays with WT and Δ*gtrR* M. tuberculosis.

Raw 246.7 macrophages were supplied at passage 4 from the Wood lab (Georgia Tech). Cells were grown in Dulbecco’s modified Eagle’s medium (DMEM) plus glutamine without pyruvate and without addition of antibiotics. Raw 246.7 macrophage cells used for this assay were between passage 6 and 10. Cells were grown to 100% confluence in Greiner CellStar tissue culture-treated 6-well plates. Macrophage cells were washed with sterile Dulbecco’s PBS (DPBS), and fresh medium was added prior to infection with M. tuberculosis. WT M. tuberculosis and Δ*gtrR* M. tuberculosis were grown to an OD_600_ of ~1 as described above. WT M. tuberculosis was diluted 1:10 into fresh 7H9/ADS medium with 25 μM hemin chloride, and Δ*gtrR* M. tuberculosis was diluted into 7H9/ADS medium with 25 μM hemin chloride and 5 μg/mL ALA. Diluted WT and Δ*gtrR* M. tuberculosis cells were grown for ~7 days until an OD_600_ of ~1 was reached. M. tuberculosis cells were depleted of heme for 2 days by washing with 7H9 medium and resuspension in fresh 7H9/ADS without hemin chloride. For infection, M. tuberculosis cells were washed and resuspended in sterile DPBS at an OD_600_ of 4 (100× multiplicity of infection [MOI]). M. tuberculosis cells were added to macrophage cells at an MOI of 10 (OD_600_ of 0.04) for 3 h and incubated at 37°C in 5% CO_2_. Three wells of macrophages treated with PBS were used as controls. After 3 h, extracellular M. tuberculosis was washed away using three washes with sterile DPBS, and fresh DMEM was added with 40 μg/mL kanamycin to kill any extracellular M. tuberculosis. Cells were incubated for 24 h at 37°C in 5% CO_2_. Cells were washed with sterile DPBS and lysed with 500 μL sterile filtered 0.05% SDS in DPBS for 5 min at room temperature. To aid in lysis, SDS was pipetted up and down. Then, 400 μLs of lysate was pelleted, washed with PBS, and resuspended in 200 μL PBS. Next, 100 μL of each sample was added in duplicate to a 96-well plate along with 20 μL of sterile filtered 5 mg/mL MTT in water. The MTT mixture was incubated at 37°C for ~20 h. Empty wells in the plates were filled with sterile water, and the plates were sealed with parafilm to decrease evaporation. After 20 h, 100 μL of 10% SDS in water was added to dissolve formazan crystals. This mixture was placed at 37°C for 4 h, and then absorbance was read at 570 nm and 690 nm. The 690-nm absorbance was subtracted as background. Readings for macrophage-only wells were greater than 5-fold lower than any M. tuberculosis-infected well readings. These values were subtracted as background from wells infected with M. tuberculosis. For the M. tuberculosis preinfection MTT assay, cells were resuspended at an OD_600_ of 4 in sterile DPBS, and 16 μL of cells was diluted to 200 μL in sterile DPBS and added in duplicate 2 × 100 μL to a 96-well plate. Then, 20 μL of sterile filtered 5-mg/mL MTT was added, and the cells were incubated 37°C for 20 h. SDS was added for 4 h to dissolve formazan, and then the absorbance was read as described above for the macrophage-treated cells.

### Macrophage infection assays with WT M. tuberculosis expressing heme sensors.

For infection assays with WT M. tuberculosis expressing heme sensor, macrophage cells were grown as described in the previous section with the exception that cells were passaged onto 35-mm glass-bottom MatTek plates to allow for imaging via microscopy. WT M. tuberculosis and WT M. tuberculosis expressing HS1, HS1-M7A, and HS1-M7A/H102A was grown in 7H9/ADS with 40 μg/mL kanamycin sulfate. After infection for 3 h and washing away of extracellular M. tuberculosis with 3 sterile DPBS washes, macrophages were suspended in DMEM medium (as described in the previous section) supplemented with 40 μg/mL kanamycin sulfate and 75 μg/mL hygromycin B. Fluorescence of WT M. tuberculosis was measured before infection as previously described. At each time point after the 3-h infection, cells were imaged via microscopy on a Zeiss LSM 710 microscope using a 64× oil objective with Texas Red (for mKATE) and GFP filters. Macrophage cells were then scraped from the MatTek dishes, and the fluorescence of WT M. tuberculosis expressing sensors was measured in a BioTek Synergy MX plate reader as reported above. Macrophage cells infected with WT M. tuberculosis cells without sensor were measured for fluorescence in each channel and subtracted as background. For microscopy images, Fiji (70) was used to subtract the background, merge the eGFP and mKATE channels, and calculate the eGFP/mKate ratios. The images shown are representative of at least 10 unique regions on each MatTek dish.
