# Supplementary material for: Exogenously Scavenged and Endogenously Synthesized Heme Are Differentially Utilized by Mycobacterium tuberculosis
Source: Microbiol Spectr. 2022 Sep 28;10(5):e03604-22. doi: 10.1128/spectrum.03604-22 (PMC9604157; doi:10.1128/spectrum.03604-22)
Supplement: Supplemental file 1 — Supporting Figures and Tables. Download spectrum.03604-22-s0001.pdf, PDF file, 0.8 MB [file spectrum.03604-22-s0001.pdf]

Supporting Information

Exogenously scavenged and endogenously synthesized heme is differentially utilized by *Mycobacterium tuberculosis*.

Rebecca K. Donegan, Yibo Fu, Jacqueline Copeland, Stanzin Idga, Gabriel Brown, Owen F. Hale, Avishek Mitra, Hui Yang, Harry A. Dailey, Michael Niederweis, Paras Jain*, and Amit R. Reddi*

Supplemental Figures 1 – 5.
Supporting Tables 1 – 3.

Supplemental Figures.

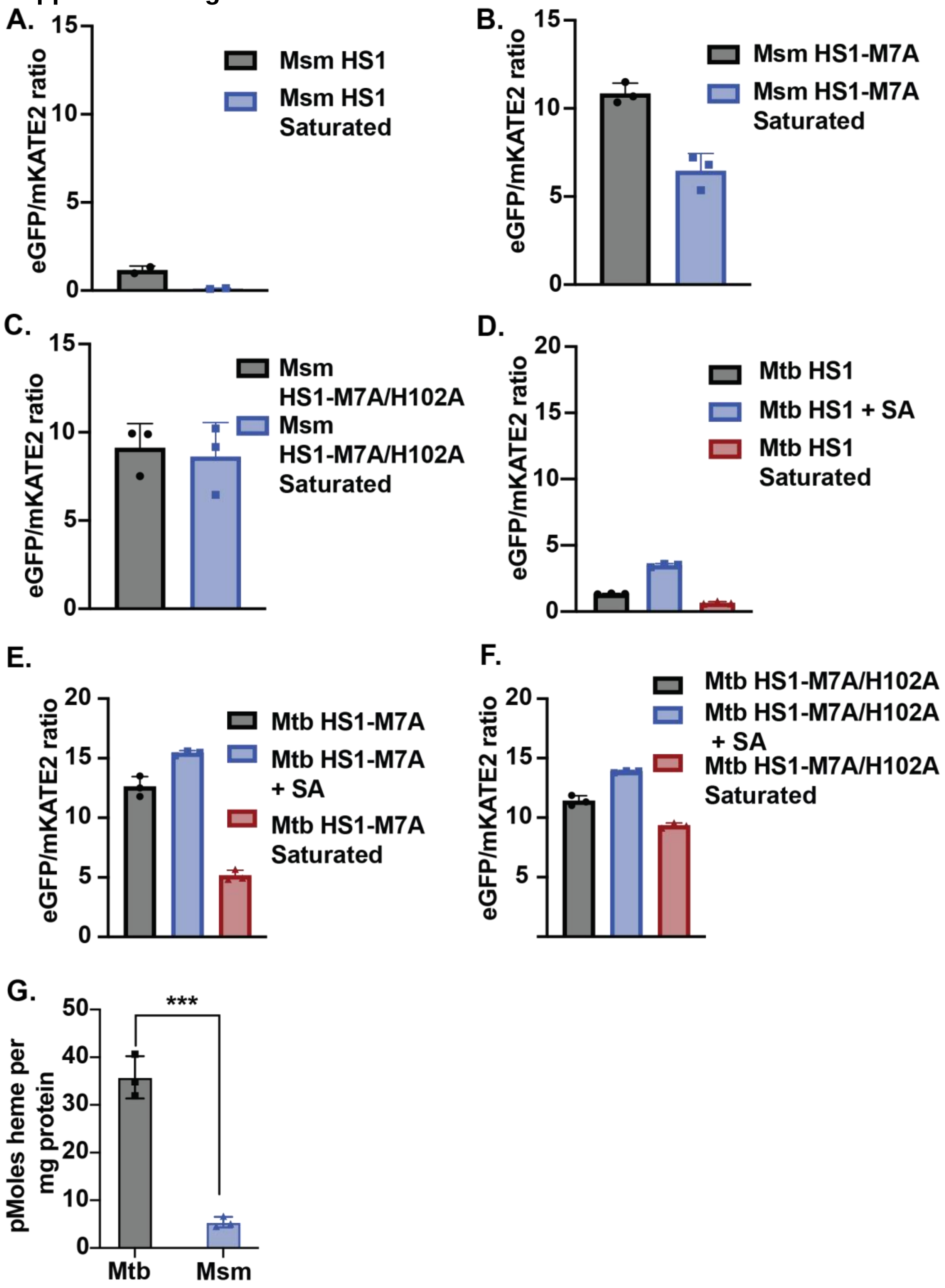

Supplemental Figure 1. In situ sensor calibration in WT Msm and WT Mtb. a. WT Msm HS1, **b.** WT Msm HS1-M7A and **c.** WT Msm HS1-M7A,H102A before and after permeabilization with sodium citrate and Triton X-100 followed by saturation with 50 µM hemin.. **D.** WT Mtb HS1 **E.** WT Mtb HS1-M7A, and **F.** WT Mtb HS1-M7A,H102A with no treatment, treatment with 500 µM SA or after permeabilization with sodium citrate and Triton X-100 and saturation with 50 µM hemin. **G.** Concentration of total heme in WT Msm and WT Mtb lysates. Data in all panels represent the mean ± S.D. (error bars) for n=3 except for panel **d.** where n=2. In panel **G,** the statistical significance was assessed by using a two-tailed unpaired t-test. ***P-value = 0.0003.

A.

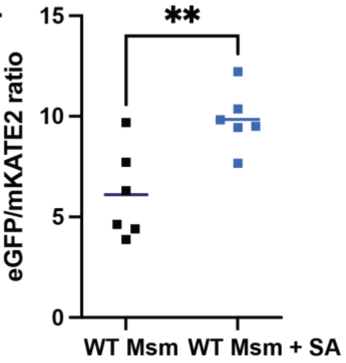

B.

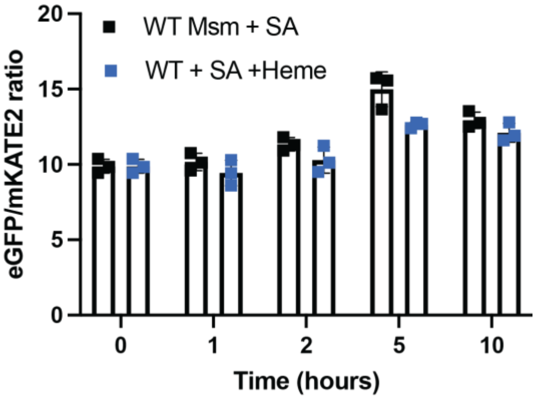

Supplemental Figure 2. Exogenous heme bioavailability in WT Msm. A. WT Msm treated overnight with 500 µM SA (WT SA, blue squares) has reduced labile heme compared to untreated WT Msm (purple squares). Data is from two independent trials. **B.** Addition of heme does not increase labile heme as measured by the HS1-M7A sensor in WT Msm treated with 500 µM SA. Data shown is representative of two independent trials. In panel **A**, statistical significance was assessed by an unpaired two-tailed student's t-test and p value = 0.0069.

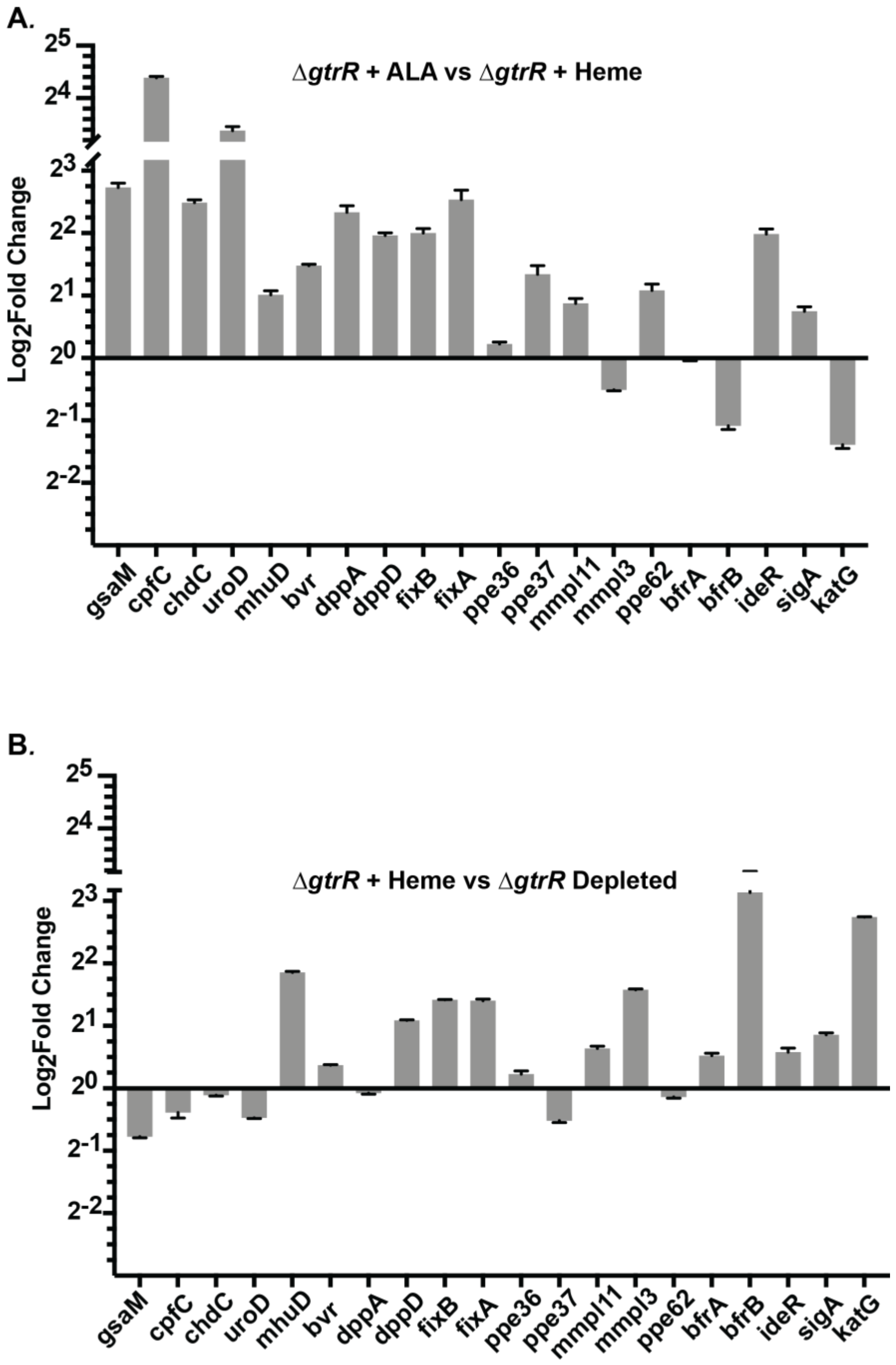

Supplemental Figure 3. Pairwise comparisons of transcript level fold change between different growth conditions of Mtb. All data are from two biological replicates with error bars representing +/- S.D. **A.** Log_2 fold change between Δ*gtrR* Mtb grown in 5 μg/mL ALA versus Δ*gtrR* Mtb grown in 25 μM heme for 3 days **B.** Log_2 fold change between Δ*gtrR* Mtb grown in 25 μM heme vs Δ*gtrR* Mtb grown in heme and then depleted for 3 days.

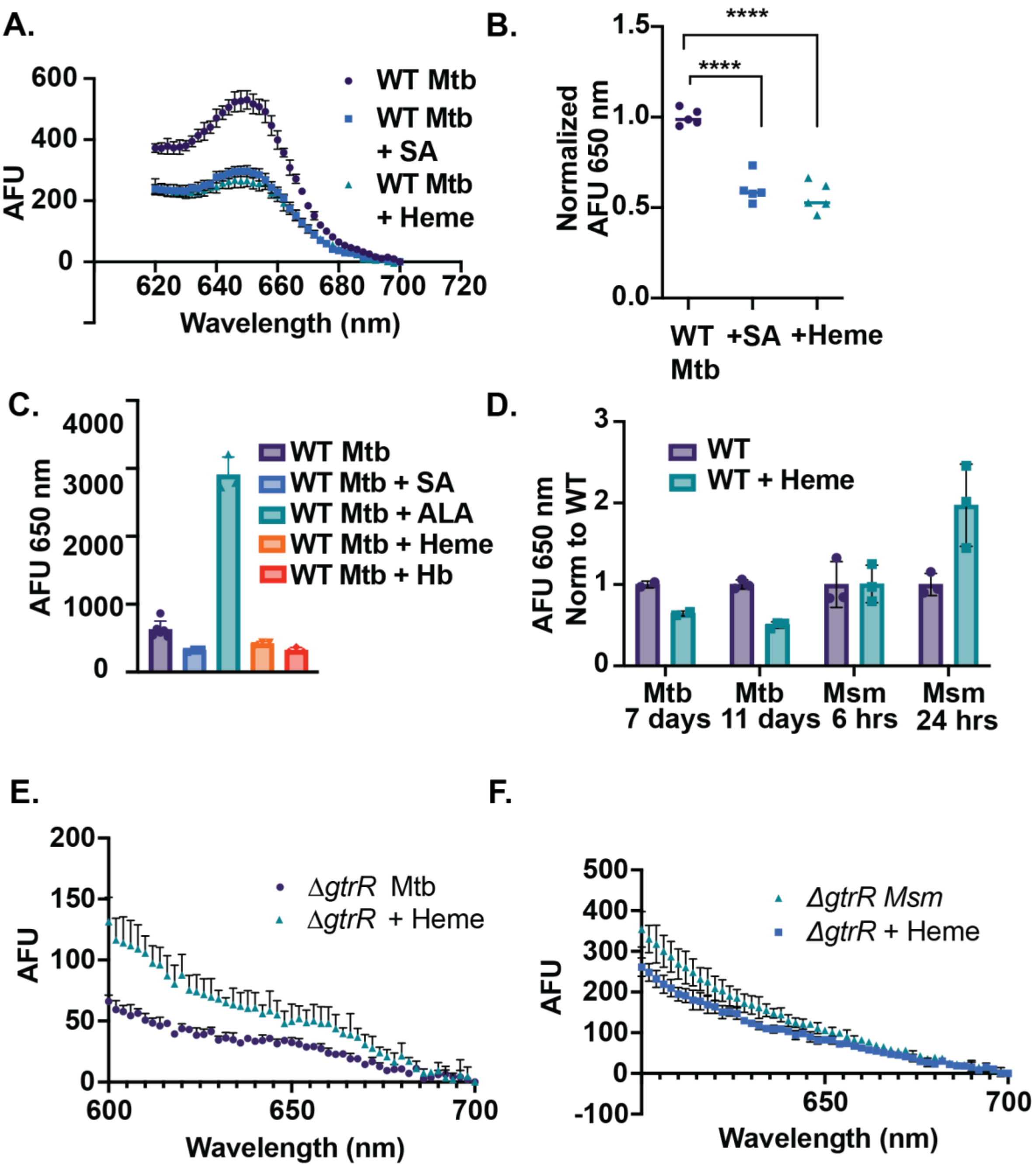

A.
AFU
600
400
200
0
620
640
660
680
700
720
Wavelength (nm)
WT Mtb
WT Mtb + SA
WT Mtb + Heme
B.
Normalized AFU 650 nm
1.5
1.0
0.5
0.0
****
****
WT Mtb
+SA
+Heme
C.
AFU 650 nm
4000
3000
2000
1000
0
WT Mtb
WT Mtb + SA
WT Mtb + ALA
WT Mtb + Heme
WT Mtb + Hb
D.
AFU 650 nm Norm to WT
3
2
1
0
WT
WT + Heme
Mtb 7 days
Mtb 11 days
Msm 6 hrs
Msm 24 hrs
E.
AFU
200
150
100
50
0
600
650
700
Wavelength (nm)
ΔgtrR Mtb
ΔgtrR + Heme
F.
AFU
500
400
300
200
100
0
-100
650
700
Wavelength (nm)
ΔgtrR Msm
ΔgtrR + Heme

Supplemental Figure 4. Iron-free porphyrin in *M. smegmatis* and *M. tuberculosis*. A. Representative iron-free porphyrin fluorescence spectra and (**B**) quantification of the emission peak at 650 nm from replicates in two independent trials are shown n=5. **C.** Iron-free porphyrin (FPs) levels of WT Mtb control (WT Mtb) or treated with 500 µM SA, 5 µg/mL 5-ALA, 25 µM Heme or 6.25µM Hb for 11 days. Error bars shown are S.D. Spectra are average of spectra from 3 biological replicates. **D.** Iron-free porphyrin fluorescence at 650 nm of WT Mtb and WT Msm treated with 25 µM Heme for given times. **E.** Background porphyrin spectra in ΔgtrR Mtb depleted of ALA with and without heme. **F.** Background porphyrin spectra in ΔgtrR Msm depleted of ALA with and without heme. In panel **B.**, the statistical significance was assessed by a one-way ANOVA with Dunnett's post hoc test using WT Mtb as control. In panel **B**, **** $p < 0.0001$.

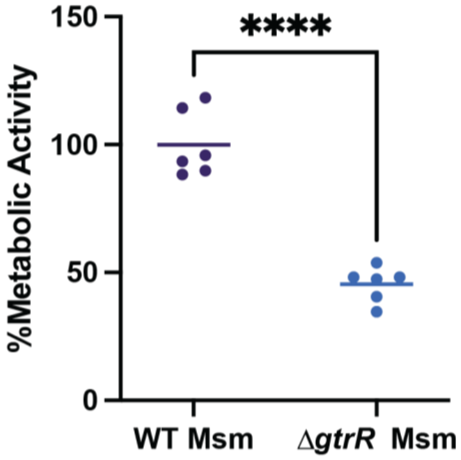

Supplemental Figure 5. Role of heme synthesis in *M. smegmatis* in a macrophage infection model. Formazan absorbance reported as % metabolic activity of WT Msm and Δ*gtrR* Msm, normalized to WT Msm as 100% isolated from RAW 246.7 macrophages after 4 hours of infection from 2 independent trials. WT Mtb n=6 and ΔGtrR Mtb n=6. In panel. Statistical significance was assessed by an unpaired two-tailed student's t-test and p value < 0.0001.

Supplemental Tables

Table 1: Heme biosynthesis enzymes and abbreviations found in the oxygen dependent pathways. Colors correspond to pathways in Figure 1.

Enzyme name	Old abbreviation(s)	New abbreviation	Rv number (Mtb)
ALA synthase	HemA	AlaS	N/A
Glu-tRNA reductase	HemA/GtrA	GtrR	Rv0509
GSA mutase	HemL/GSAM	GsaM	Rv0524
ALA dehydratase/PBG synthase	HemB/ALAD/PBGS	PbgS	Rv0512
PBG deaminase/HMB synthase	HemC/HMBS/PBGD	HmbS	Rv0510
URO synthase	HemD/UROS	UroS	Rv0511
URO decarboxylase	HemE/UROD	UroD	Rv2678c
Coproporphyrinogen decarboxylase	HemF/CPOX	CgdC	N/A
Protoporphyrinogen oxidase	HemY	PgoX	N/A
Protoporphyrin ferrochelatase	HemH	PpfC	N/A
Coproporphyrinogen oxidase	HemY	CgoX	Rv2677c
Coproporphyrin ferrochelatase	HemH/HemZ	CpfC	Rv1485
Coproheme decarboxylase	HemQ	ChdC	Rv2676c

Table 2. Primers used in this study

Primer Name	Sequence
HS1 Forward	GGAATTCGATATCAAGCTTTTTAAGGAGATATACATATGCAC ATGGTATCGGAACTGATC
HS1 Reverse	CCCCCCCCCCGACGTCAGGTGGCTAGCTTCATTTATACAG TTCATCCATACCCAAAGTG
pYUB1872 Forward	ATGTATATCTCCTTAAAAAGCTTGATATCGAATTCCTGC
pYUB1872 Reverse	CGATAGCTAGCCACCTGACGTCGGGG
HS1 internal	AGGTGGCGGTCACTTGATTTGC
sacB out_pYUB1471	CGGCAGGTATATGTGATGGG
Hyg_out_pYUB1471	AACTGCTCGCCTTCACCTTC
gtrR (Rv0509) LL	TTTTTTTTCCATAAATTGGCATGGTGTTCGCCGTTACCA
gtrR (Rv0509) LR	TTTTTTTTCCATTTCTTGGGTACCAGATTCGGGATGTCTGATG TCTCACTGAGGTCTCTTACGATGCGACACCCCGAAG
gtrR (Rv0509) RL	TTTTTTTTCCATAGATTGGGATGGTATTCACTCGGTGTCCGA GTGTCTGGTCTCGTAGGCGGATTCGACGCTGAAAGT
gtrR (Rv0509) RR	TTTTTTTTCCATCTTTTGGGCACCGGCTCTAACGTCTCG
gtrR (Rv0509) KoC_UP	CCTCTTTCGGGCTTTCCGTATTG
gtrR (Rv0509) KoC_DN	GCGACAGCTCCTCGAAGACAC
gtrR (MSMEG_0952) LL	TTTTTTTTCCATAAATTGGGTGCCGGGCCAGATCTTGTC
gtrR (MSMEG_0952) LR	TTTTTTTTCCATTTCTTGGCAGCACGCTCACGGCTTCATC
gtrR (MSMEG_0952) RL	TTTTTTTTCCATAGATTGGACTGAGTAGAGCTTGGCAAACAA C
gtrR (MSMEG_0952) RR	TTTTTTTTCCATCTTTTGGGACCTGCCGGGTCATTTCTC
gtrR (MSMEG_0952) KoC_UP	CGCGGTGACCAGCCAGAC
gtrR (MSMEG_0952) KoC_DN	GCCGAAGGTGGCCAGACTC
Kan pro out	TAATCGCGGCCTCGAGCAAG

G13 Promoter seq primer	TGGTCGATACCAAGCCATTTCC

Table 3. Primers used for qPCR in this study.

Gene Name	Rv Number	Forward Primer	Reverse Primer
gtrR	Rv0509	ACCGCCTGGCTAATGTCCTG	GCATGCCCAAGTCGCATATC
gsaM	Rv0524	TTGTGCCACACCAGATTTCG	CAGCATGGCATGAAAGAACG
cpfC	Rv1485	CGTTCCTGGAGAACGTTACC	ACGGCATCTTCTACATACGG
chdC	Rv2676c	TTTGTGCGGTCTTACGAGTG	ACGCCAGGATCCATTCGTAG
uroD	Rv2678c	ATTACCCTGCAGCCGATACG	TACCGGTTGAATCGCTTGTG
mhuD	Rv3592	CCTCGGCTTTCAGCTGTTAC	TCAAGCACGACCTCGAATTCC
BVR	Rv2074	GCGATGGTCAACACCACTAC	GGGTCGAAGGTGAAACCTAC
dppA	Rv3666c	CCCGTCGATGATCGAGTTTC	CGACGACACTGATGTAATCC
dppD	Rv3663c	GGGACTCCCGGTAAATCTTG	GTGGAGTGGTGGTGGAATC
fixB	Rv3029c	AAGCGCTACAGATTCGGGAG	ATGCCGTCGTCCTTTAGGTG
fixA	Rv3028c	TGCCGCCAAGATCTACGTC	GACACCCACTCCACCTTCTC
ppe36	Rv2108	CACGTTGCTGGAGTCGTATAG	TGCCTTCAACACTGTGGTCTG
ppe37	Rv2123	CGACCCGACCAAATTGATCC	GGCGAGAAACGTGAAGACTG
mmpL11	Rv0202c	CCTGCCTATCATTCTGATGG	AGGATGAACAGGGAGTAGTC
mmpL3	Rv0206c	CGGCGAATATGTGGCAAGAG	AGGCCGTGGATTGAATCCAG
ppe62	Rv3533c	GAAACGGCGGCGCAATAAAC	GACCAGCCGGTATTTCTGAC
bfrA	Rv1876	ACGACGTGTTGAATCGTCTC	AAGCTCCTCTCCTAGCTTGT
katG	Rv1908c	GACAAGGCGAACCTGCTTAC	TCCCAGGTGATACCCATGTC
ideR	Rv2711	GGCTGGTCAAGGTGCTCAAC	CCCTGAACGTGCTCGGTAAG
bfrB	Rv3841	GACCTTCGTGTCGAAATTCC	GATCTGTTCCTGCAAGAACC
sigA	Rv2703	CAGCTGATGACCGAGCTTAG	CCTGGATCAGGTCGAGAAAC
16s rRNA	rrs	CACTGGGACTGAGATACGGC	CTCCACCTACCGTCAATCCG
